# Application of circular consensus sequencing and network analysis to characterize the bovine IgG repertoire

**DOI:** 10.1186/1471-2172-13-52

**Published:** 2012-09-14

**Authors:** Peter A Larsen, Timothy P L Smith

**Affiliations:** 1 Genetics and Breeding Unit, United States Meat Animal Research Center, ARS, USDA, State Spur 18D, 68933, Clay Center, NE, USA; 2 Present address: Department of Biology, Duke University, Box 90338, 27708, Durham, NC, USA

**Keywords:** Antibody diversity, *Bos taurus*, SMRT sequencing, Immunoglobulin G

## Abstract

**Background:**

Vertebrate immune systems generate diverse repertoires of antibodies capable of mediating response to a variety of antigens. Next generation sequencing methods provide unique approaches to a number of immuno-based research areas including antibody discovery and engineering, disease surveillance, and host immune response to vaccines. In particular, single-molecule circular consensus sequencing permits the sequencing of antibody repertoires at previously unattainable depths of coverage and accuracy. We approached the bovine immunoglobulin G (IgG) repertoire with the objective of characterizing diversity of expressed IgG transcripts. Here we present single-molecule real-time sequencing data of expressed IgG heavy-chain repertoires of four individual cattle. We describe the diversity observed within antigen binding regions and visualize this diversity using a network-based approach.

**Results:**

We generated 49,945 high quality cDNA sequences, each spanning the entire IgG variable region from four *Bos taurus* calves. From these sequences we identified 49,521 antigen binding regions using the automated Paratome web server. Approximately 9% of all unique complementarity determining 2 (CDR2) sequences were of variable lengths. A bimodal distribution of unique CDR3 sequence lengths was observed, with common lengths of 5–6 and 21–25 amino acids. The average number of cysteine residues in CDR3s increased with CDR3 length and we observed that cysteine residues were centrally located in CDR3s. We identified 19 extremely long CDR3 sequences (up to 62 amino acids in length) within IgG transcripts. Network analyses revealed distinct patterns among the expressed IgG antigen binding repertoires of the examined individuals.

**Conclusions:**

We utilized circular consensus sequencing technology to provide baseline data of the expressed bovine IgG repertoire that can be used for future studies important to livestock research. Somatic mutation resulting in base insertions and deletions in CDR2 further diversifies the bovine antibody repertoire. In contrast to previous studies, our data indicate that unusually long CDR3 sequences are not unique to IgM antibodies in cattle. Centrally located cysteine residues in bovine CDR3s provide further evidence that disulfide bond formation is likely of structural importance. We hypothesize that network or cluster-based analyses of expressed antibody repertoires from controlled challenge experiments will help identify novel natural antigen binding solutions to specific pathogens of interest.

## Background

The vertebrate immunoglobulin (Ig) locus has evolved to generate a large potential repertoire of antigen binding sites capable of mediating response to a plethora of antigens. In many species (including cattle), the actual expressed diversity generated relative to genomic potential has not been thoroughly described because the sizeable number of potential unique specificities (e.g. ~1 x 10^7^) made it difficult to perform adequate surveys of the expressed repertoire. Recent advances in high-throughput sequencing technologies permit the sequencing of antibody repertoires at previously unattainable read-lengths and depths of coverage, therefore allowing researchers to better explore antibody diversity and selection within individuals [1-3]. In particular, single-molecule real-time (SMRT) circular consensus sequencing (CCS) is ideally suited for exploring the diversity of expressed antibodies because this sequencing method provides multiple reads of individual templates, resulting in higher per-base sequencing accuracy and the reduction of stochastic error [4].

**Figure 1:**
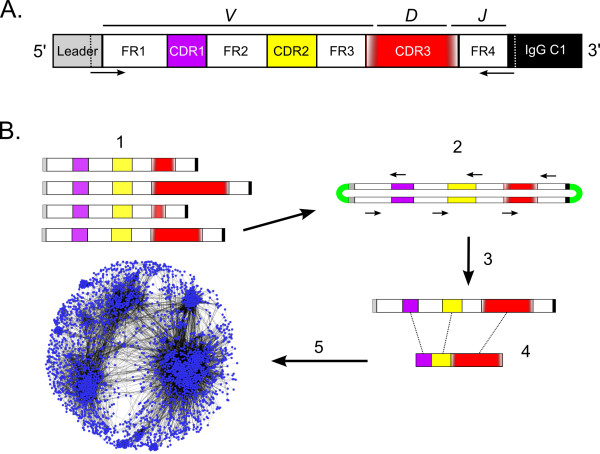
**A: Diagram of the IgH variable region.** Dashed lines identify primer binding sites used for PCR amplification of IgG cDNA transcripts. FR = framework region; CDR = complementarity determining region; C1 = constant region 1; *V*, *D*, and *J* = variable, diversity, and joining segments. Color gradations flanking CDR3 indicate junction sequence diversity. **B**: Experimental design for single-molecule real-time (SMRT) sequencing, data analysis, and visualization of IgG antigen binding regions. **1**: cDNA libraries are constructed from PCR amplicons of the variable region from expressed IgG transcripts. Differing lengths are primarily the result of variation in CDR3. **2**: SMRTbells™ are prepared for sequencing by ligating adapters to individual double-stranded amplicons. Small arrows indicate multiple passes of bound DNA polymerase around the SMRTbell™ resulting in several reads of the template. These reads are concatenated into circular consensus (CCS) sequences. **3**: CCS sequences are filtered based on quality and correct open reading frame is determined based on alignment with the conserved FR1. **4**: Antigen binding regions (CDRs1–3) are identified from translated sequences and are extracted from each sequence. **5**: Networks are constructed from all vs. all BLAST searches of thousands of antigen binding regions from each IgG repertoire.

The typical antibody molecule consists of two heavy-chains and two light-chains, each with variable (V) and constant (C) domains. Antibody diversity is generated primarily through a recombination process among a set of three families of germline gene segments that occurs during maturation of the antibody expressing cells, although additional variation can be introduced by gene conversion, nucleotide insertions/deletions, receptor editing, and somatic hyper-mutation. Figure 1 contains a schematic representation of the heavy chain protein illustrating the framework (FR), complementarity determining (CD), and C regions, and their relationship to the variable (*V*), diversity (*D*), and joining (*J*) gene segments [5,6]. Alternative combinations of *V*, *D*, and *J* segments and junctional diversity, coupled with somatic hypermutation, generate a surprising number of potential antibody sequences with at least 1 × 10^7^ unique antibody binding sites estimated for humans and *Mus*[7]. This diversity is in part due to the functional V_H_ genes present in the germlines of these species, with humans having approximately 44 *V*, 27 *D*, and 6 *J* segments and *Mus* approximately 219 *V*, 21 *D*, and 4 *J* segments [8,9]. The germline *V* segment diversity for humans and *Mus* is classified into 7 and 16 gene families, respectively [9]. In contrast, the bovine repertoire is derived from a single family of germline *V* segments that is closely related to human VH4 and murine Q-52 families [10-12]. The total number of germline *V* segments in *Bos taurus* remains unknown but is hypothesized to consist of 13 to 20 conserved segments [11,13]. Several studies have focused on *D* and *J* gene segment diversity of *Bos taurus*, and these hypothesize the presence of approximately 10 *D* and 6 *J* segments (including potential pseudogenes) within the *Bos* genome [14-16]. Interestingly, excessive CDR3 length variability (with respect to other mammalian species) has been observed in bovine Igs. This CDR3 length variability is likely associated with the limited number of functional germline *V*, *D*, and *J* segments within the *B. taurus* genome, perhaps serving to further diversify the bovine immune response [13,14,17].

Identification and analysis of the variation observed in antigen binding regions of expressed antibody sequences is of particular interest because such data will likely provide unique approaches to a number of immuno-based research areas including antibody discovery and engineering, disease surveillance, immunotherapy, and host immune response to vaccines [18-22]. It is within this framework that we examined the bovine IgG repertoire in young, apparently healthy animals. We focused first on IgG because of its central role in the adaptive immune response and because of the importance of this response to vaccination success. Moreover, future analyses of the antigen binding regions of expressed IgG transcripts using high-throughput methods may prove useful for many areas of livestock research (e.g. immune response to bacteria, parasites, and viruses). Here we present SMRT CCS data of the expressed IgG repertoires from four *B. taurus* juveniles 1 to 2 months of age. The immune systems of the individuals examined herein are expected to be relatively naïve compared to those of adults and thus provide a suitable starting point for characterizing baseline antibody diversity. We describe the diversity observed in IgG heavy-chain antigen binding regions and visualize this diversity using a network-based approach.

## Methods

### Animal samples and total RNA production

Animal procedures were reviewed and approved by the United States Meat Animal Research Center (USMARC) and National Animal Disease Center (NADC) Animal Care and Use Committees. Peripheral blood samples (10 cc) were collected from two crossbred calves (Brown Swiss × Red Angus-Simmental; Calf 1 = USMARC 20113360, Calf 2 = USMARC 20113363) and two purebred Holstein calves (Calf 3 = NADC 1478, Calf 4 = NADC 1480). All calves were approximately 1 to 2 months old at the time of sampling and blood samples were taken prior to immunization. Whole blood was centrifuged at 2000 × *g* for 15 min at room temperature and leukocytes were collected and stored at −80°C. Total RNA was isolated from leukocyte enriched samples using TRIzol® LS (Life Technologies, Grand Island, NY) following the manufactures' protocol for biological fluids. RNA pellets were resuspended in RNase-free H_2_0 and OD260/280 measurements were taken to quantify each sample.

### Primer design, cDNA synthesis, PCR, and sequencing

A complete germline genome sequence of the bovine immunoglobulin locus was not available, as existing draft genomes were produced using DNA derived from blood cells. To facilitate primer design targeting the variable region of the heavy chain of IgG mRNA, we developed a database of bovine EST sequences based on BLAST searches (blast.ncbi.nlm.nig.gov) of the bovine V_H_ region (GenBank accession numbers U55164–U55169, U55171, U55172, U55174, U55175 [10,23]) and constant regions of IgG1, IgG2, and IgG3 (GenBank accession numbers S82409, S82407, and BTU63638; [24,25]). Primers targeting the leader sequence of the V_H_ region and IgG C1 were gathered from previously published reports [15,26] and were modified based on the variation observed in the EST database. cDNA of full length immunoglobulin mRNA was synthesized using the SMARTer PCR cDNA Synthesis Kit (Clontech Laboratories, Inc., Mountain View, CA) and a 5' PCR primer specific to the 5' end of the V_H_ leader sequence (5'-CTC-SAA-GAT-GAA-CCC-ACT-GTG-3'). Subsequent PCRs of cDNA libraries targeted the IgG V_H_ region (~300–450 bp) by using primers specific to the 3' end of the IgV_H_ leader (5'-CCC-TCC-TCT-TTG-TGC-TST-CA-3') and a conserved region of the C1 domain of IgG1, IgG2, and IgG3 (5'-TTT-CGG-GGC-TGT-GGT-GGA-SG-3'). Amplicons were obtained using a high fidelity *Taq* DNA Polymerase (AccuPrime; Life Technologies, Grand Island, NY) and the following thermal profile: initial denaturation at 94°C for 2 min followed by 33 cycles of 94°C for 15 sec, 54°C for 45 sec, and 72°C for 1 min. SMRT sequencing was performed with a Pacific Biosciences RS sequencer following manufacturer's protocols for CCS. The ccs.fastq files created by the instrument's basecalling software were used for subsequent analyses.

### Quality filtering and sequence data analysis

Quality filtering of CCS cDNA sequences was performed using the Galaxy platform [27-29] to retain V_H_ sequences in which at least 97% of the bases had a quality score > 20 (1% error rate). Geneious Pro software (version 5.5.6; Biomatters Ltd.) was used to assemble and align sequence data. The length variability of CDR3 can confound correct determination of reading frame in the amplified fragments, so open reading frames were determined by aligning reads to a reference consensus sequence of the conserved FR1 region from *B. taurus* germline *V* segments (GenBank accession numbers U55164–U55169, U55171, U55172, U55174, U55175 [10,23]; Figure 1). The predicted amino acid sequences of the expressed variable regions were inferred by standard *in silico* translation of the open reading frame nucleotide sequences, and all reads with stop codons were eliminated from the dataset. The final dataset consisted of only those reads that encoded the conserved 5' terminal portion of the IgG C1 exon (including isotypes IgG1, IgG2, IgG3 [24,25,30]. Cluster analyses were performed using the CD-HIT web server [31] and descriptive statistics were calculated using Geneious Pro and Microsoft Excel 2007™ software.

### CDR identification

Several definitions exist for the term complementarity determining region (CDR), however, we use CDR to refer to the residues that form the basis of antigen interaction [32]. Multiple methods have been implemented to identify antigen binding residues within antibody sequences [9,33-36] and (depending on the classification/numbering scheme used) the boundaries of these regions can fluctuate (see Additional file 1: Table S1). Moreover, conventional CDR identification methods (e.g. the Kabat numbering system) can be difficult to implement when analyzing large datasets and can potentially exclude antigen binding region data. Several studies have indicated that structure-based methods provide a more accurate identification of CDRs in antibody sequence data [32,37,38]. Thus we utilized the structure-based automatic sequence antigen binding region identification tool known as Paratome ( http://ofranservices.biu.ac.il/site/services/services.html) [32,38] to identify CDRs within our translated IgG cDNA sequence data. We compared our results with previous analyses of bovine IgH sequence data and, for ease of comparison with other studies, we report standard CDR3 position numbers for representative sequences using the International Immunogenetics Information System (IMGT) naming convention [9].

### Network analyses

We extracted and concatenated amino acid residues of the complete antigen binding region (CDRs1–3; as identified by Paratome) of individual IgG transcripts for each repertoire examined. All vs. all BLAST searches were performed on the CDR databases using default blastp parameters and an *E*-value of 1x10^-8^. BLAST results were used to construct networks with the Cytoscape software platform (version 2.8; http://www.cytoscape.org) using the BLAST2SimilarityGraph plugin ( http://transclust.cebitec.uni-bielefeld.de) and the sum of all hits similarity function for edge weights. The edge-weighted spring embedded algorithm was used to visualize networks and connectivity analyses were performed using the NetworkAnalyzer plugin [39].

## Results

**Figure 2:**
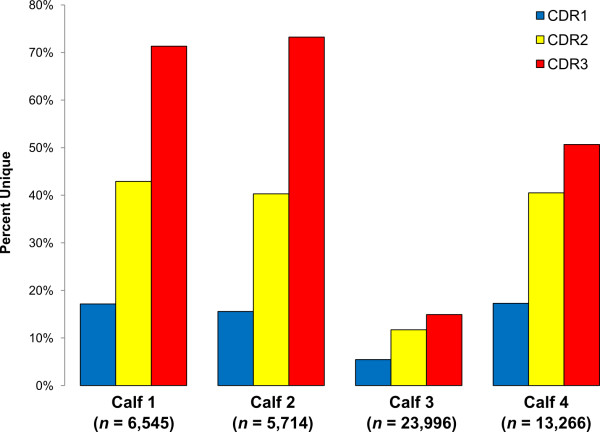
Percentage of unique CDR1, 2, and 3 amino acid sequences per IgG repertoire.

Circular consensus sequencing resulted in a total of 409,164 sequences. Filtering based on quality score values reduced the set to 70,610 sequences, alignment of this reduced set to bovine consensus FR1 resulted in 68,169 sequences, elimination of sequences with stop codons and confirmation of residues encoding IgG C1 in the predicted reading frame resulted in 49,945 (12% of starting sequences) of the entire IgG V_H_ region available for analysis. We performed preliminary Paratome analyses of our translated IgG sequence data and confirmed the tool's efficiency by comparing the results with previously identified CDRs of bovine immunoglobulins (Additional file 1: Table S1). Subsequent Paratome analyses were performed on each of the four IgG repertoires examined and complete antigen binding motifs were identified for 49,521 sequences (Calf 1 = 6,545; Calf 2 = 5,714, Calf 3 = 23,996; Calf 4 = 13,266). Percentages of unique CDR 1, 2, and 3 regions (per repertoire) are shown in Figure 2.

### Overall amino acid composition and length variation in CDR regions

**Figure 3:**
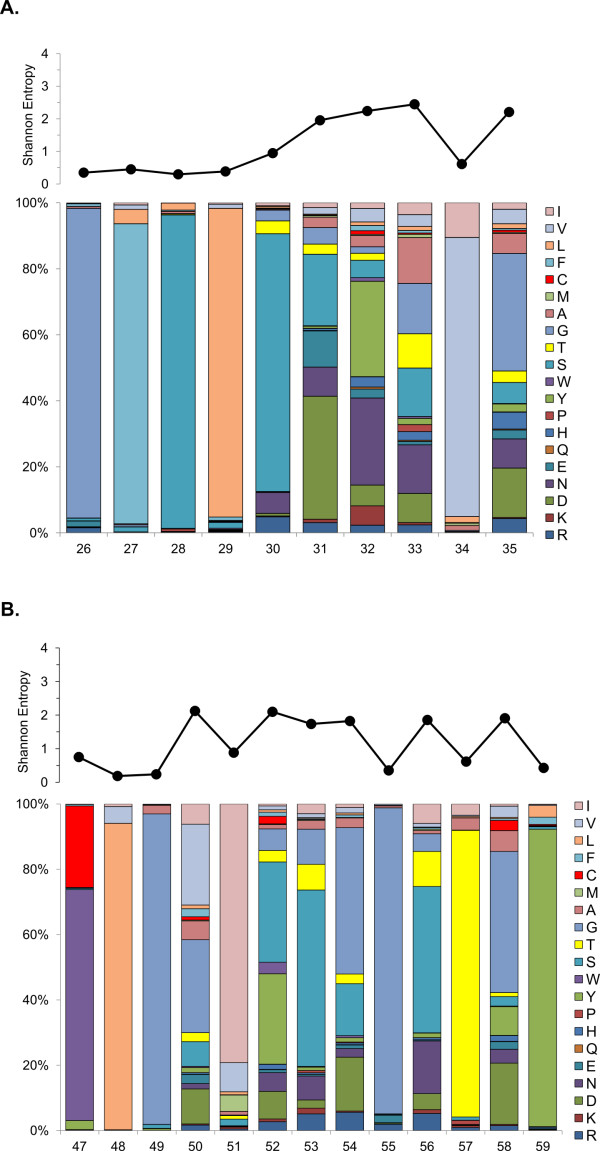
**Shannon entropy values (top panels) and amino acid frequencies (bottom panels) of unique CDR1 (A) and CDR2 (B) sequences identified by Paratome from all repertoires examined.** The most common CDR2 length class (13 amino acids) is shown. *X*-axis values are amino acid positions from the start of framework region 1 with each bar representing 100% of the amino acids identified at that position. Amino acids are grouped according to hydrophobicity with charged amino acids at the bottom and hydrophobic amino acids at the top.

All CDR1s identified by Paratome were 10 amino acids in length. Shannon entropy plots revealed increased diversity at positions 31, 32, 33, and 35 (Figure 3A), roughly corresponding with traditional definitions of the bovine IgH CDR1 using the Kabat numbering convention [12,33]. Within unique CDR1s (*n* = 4,100), six amino acids accounted for 68.5% of all residues: serine (22.5%), glycine (15.6%), leucine (10.6%), valine (10.2%), and phenylalanine (9.6%). Much of this distribution can be attributed to the observation that positions 28 and 30 are predominantly serine, position 26, 27, 29 and 34 are nearly always glycine, phenylalanine, leucine, and valine, respectively. Thus, the Paratome defined CDR1 is divided into two sub-regions, with a conserved sequence of alternating polar and non-polar residues in the proximal region, followed by a region of much higher variability.

The CDR2 region had higher overall diversity than CDR1, reflected in the higher percentage of sequences observed that were unique (Figure 2). Approximately 91% of unique CDR2s identified by Paratome were 13 amino acid residues in length (*n* = 10,609), with the remaining 9% (*n* = 1,030) ranging in length from 10–18 AAs. Shannon entropy plots of amino acid variation of unique CDR2s 13 residues in length revealed highly conserved residues at positions 48, 49, 55, 57, and 59 (Figure 3b). For the group of CDR2s that were 13 amino acids in length, six amino acids accounted for 72.9% of all residues. The top four amino acids, glycine (25.3%), serine (12.4%), tyrosine (10.4%), and threonine (9.1%), are from the uncharged polar class and are overrepresented (57.2% of residues) compared to the average mammalian protein composition (approximately 23% total for these four amino acids [40]). This overrepresentation stems from the observation that three of the five highly conserved residues are glycine, threonine, or tyrosine. The nonpolar amino acids leucine (7.9%), and isoleucine (7.8%) round out the top six, but both of these are represented approximately the same as the average among mammalian proteins. Glutamine, another uncharged polar amino acid, was the most under-represented amino acid within both CDR1 and CDR2 accounting for 0.2% and 0.1% of all residues, respectively, compared to an average 4% among mammalian proteins.

**Figure 4:**
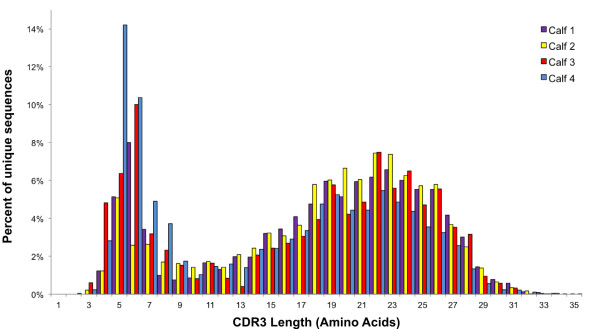
**Length distribution of unique CDR3 amino acid sequences (2–35 residues) identified using Paratome for the four individuals examined.** An additional 19 CDR3 sequences greater than 35 residues (0.036% of all reads) are shown in Additional file 1: Table S1.

**Figure 5:**
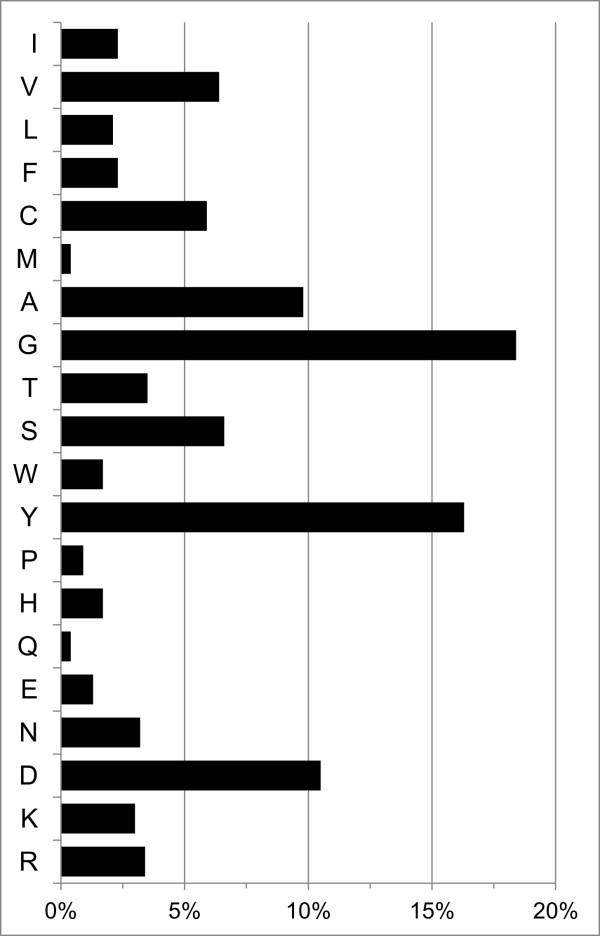
**Amino acid frequencies of unique CDR3 sequences (*n*** **= 19,039) from the expressed IgG repertoires of four individuals of *Bos taurus*.**

**Figure 6:**
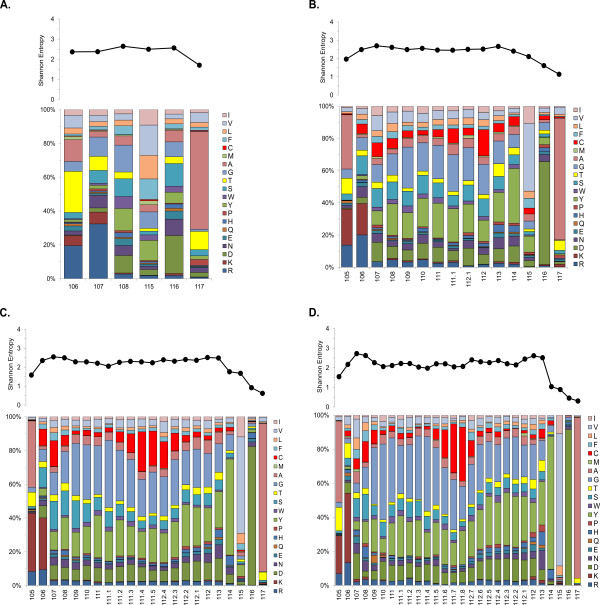
**Shannon entropy values (top panels) and amino acid (AA) frequencies (bottom panels) of unique CDR3 sequences of representative lengths from all repertoires examined. A**: CDR3 lengths of 6 AAs (*n* = 985); **B**: CDR3 lengths of 15 AAs (*n* = 523); **C**: CDR3 lengths of 22 AAs (*n* = 1,363); **D**: CDR3 lengths of 28 AAs (*n* = 524). X-axis values follow the IMGT numbering standard for CDR3 with each bar representing 100% of the amino acids identified at that position. Amino acids are grouped according to hydrophobicity with charged amino acids at the bottom and hydrophobic amino acids at the top.

The length distribution of the bovine IgG CDR3 repertoire varied from 2 to 62 amino acids and a bimodal distribution was observed, with peaks present at 5–6 and 22–23 amino acids (Figure 4). There was a substantially higher percentage of unique sequences for Calves 1 and 2, with 71.8% and 73.7% of the CDR3s identified respectively, compared to Calves 3 and 4 (13% and 52%, respectively, Figure 2). Analysis of amino acid frequencies for unique CDR3s across all individuals (*n* = 19,039) revealed that alanine, aspartic acid, glycine, and tyrosine accounted for 55% of the residues in expressed bovine CDR3s (Figure 5). We gathered unique CDR3 sequences of four representative length classes (6, 15, 22 and 28 amino acids; Figure 6) from all individuals and examined amino acid usage at each CDR3 position. CDR3 positions 116–117 were highly conserved in longer size classes (Figure 6B–D) and are attributed to the donation of aspartic acid and alanine residues from one commonly used germline *J* segment [15]. The number of cysteine residues within CDR3s was positively correlated with length (R^2^ = 0.73; Additional file 1: Figure S1) and cysteine residues were centrally located in CDR3s of all size classes (R^2^ = 0.95; Additional file 1: Figure S2). Nineteen excessively long CDR3s (32–62 amino acids) were identified within the IgG repertoires (Additional file 1: Table S2).

### Patterns of antigen binding region diversity

**Figure 7:**
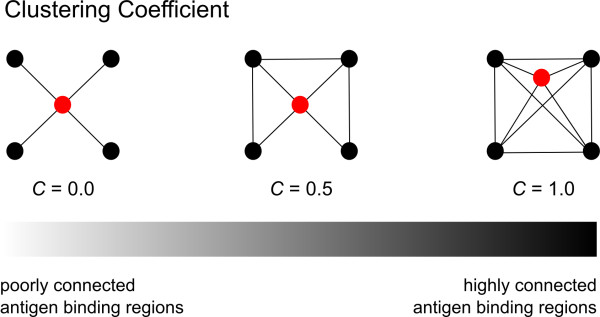
**Diagram of the clustering coefficient (*C*) used to measure network connectivity (modified from Ravasz *et al. *2002**[54]**).***C* ranges from 0 to 1 for poorly and highly connected networks, respectively.

**Figure 8:**
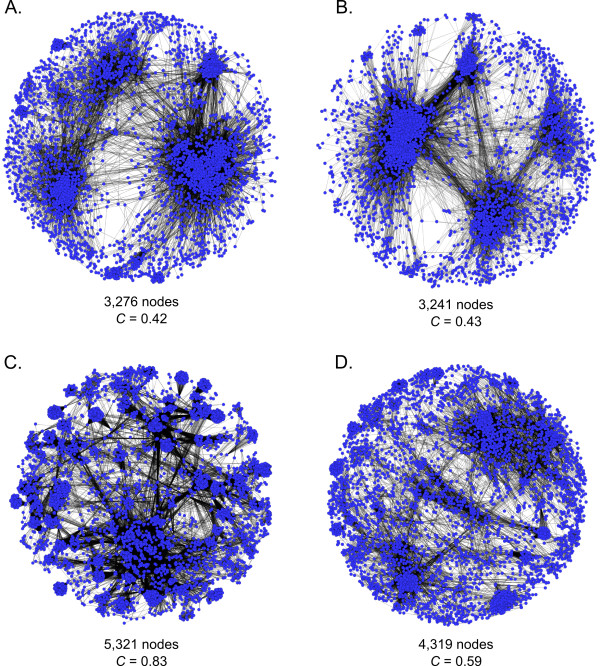
**Core antigen binding repertoire networks for each individual examined.** Number of nodes equals complete antigen binding regions (CDRs 1, 2, and 3) extracted from IgG sequences using Paratome.

We performed network analyses on concatenated antigen binding motifs in order to visualize patterns within the IgG repertoires of the four individuals examined (CDRs1–3; see Figure 1). Measures of connectivity for each network were assessed using the clustering coefficient [41] (Figure 7). Antigen binding networks having clustering coefficient (*C*) values close to 0 indicate poorly connected nodes, whereas networks exhibiting values close to 1 have highly connected nodes. Core networks of the four IgG repertoires are shown in Figure 8 and complete networks are shown in Additional file 1: Figures S3–S6. For each core network the number of nodes represents the number of antigen binding motifs connected with *E*-scores less than or equal to 1 × 10^-8^. Calves 1 and 2 displayed similar network topologies and *C* values, with both calves displaying four primary sub-clusters (Figure 8A, B). Core network topologies and associated *C* values of the repertories for Calves 3 and 4 indicate lower antigen binding diversity when compared with Calves 1 and 2 (Figure 8C, D). The network of Calf 3 was highly connected and exhibited the highest *C* value (0.83) of the four repertoires.

## Discussion

Circular consensus sequencing is a novel sequencing-based approach that allows for exploration of the diversity of expressed antibody repertoires within individuals. This is especially true for studies of the bovine Ig V_H_ region, because the 300–450 bp template length is ideal for CCS with the current version of the chemistry for the RS instrument (C2 chemistry) as it allows for the polymerase to read both strands of the molecule multiple times during sequencing (Figure 1). Other next generation sequencing technologies are limited to reading only a portion of the full antigen recognition sequence due to short read length, and/or have systematic error that can create false diversity or force analyses to “collapse” what might be true variation because it is indistinguishable from sequence error. The SMRT system has a stochastic error profile, so even though each pass of the polymerase has approximately 85% accuracy, the consensus sequence following repeated passes of both strands has high data quality and appears essentially free of systematic error [4], permitting accurate identification of molecules that differ only by a small number of nucleotide variants introduced during the recombination events associated with B cell maturation. Future improvements in read length will serve to increase the efficiency of obtaining high quality single molecule sequences by increasing the number of times each strand is read and decreasing the impact of the relatively high per-base error rate of the technology.

### Variability of bovine antigen binding regions

Sequence data of the expressed antigen binding region of the bovine IgG repertoire are comparable with previous analyses of the amino acid variability observed in mammalian CDRs [42,43] (Figures 3, 5, 6). For example, hydrophobicity of regions within IgH CDR3 is a conserved feature and is important for antigen interaction [7]. Data from bovine IgG CDR3 are consistent with this observation in that usage of the 10 most hydrophobic amino acid residues was 52.8%, compared with 54%, 44.4%, and 48.6% in humans, mouse, and bats respectively [43,44]. Moreover, tyrosine and glycine were over-represented and accounted for approximately 35% of the amino acids of CDR3. The prevalence of these residues in antigen binding regions is well documented [43,45] and it is hypothesized that tyrosine helps to stabilize antibody/antigen interactions and glycine provides conformational flexibility for antigen binding [45].

Cysteine residues within CDRs are of structural significance because disulfide bond formation serves to stabilize CDR regions and restrict CDR3 loop flexibility [46,47]. We found that cysteine residues occur at approximately 5.9% in expressed bovine IgG CDR3s, a greater frequency when compared to similar data from human and *Mus*[43] (1.21% and 0.35%, respectively). Moreover, our data indicate that the presence of cysteine residues was positively correlated with CDR3 length (*R*^2^ = 0.73; Additional file 1: Figure S1) and that these residues are centrally located in CDR3 sequences (R^2^ = 0.95; Additional file 1: Figure S2). The trend of increasing cysteine residues with length was relatively constant until CDR3s of approximately 32 amino acids, however, this pattern deviated at CDR3s of approximately 45 AAs and fluctuated from 2 to 6 cysteine residues (Additional file 1: Figure S1). This result might indicate a structural threshold within bovine CDR3s and we recommend additional analyses (e.g. X-ray crystallography) of bovine Igs to formally test this hypothesis. Overall, the patterns of bovine CDR3 cysteine residue usage observed in our data agree with those previously identified in *Bos taurus* as well as other vertebrates [42,47-49]. Collectively, these studies suggest that disulfide bond formation is important for the folding of exceedingly long immunoglobulins.

Interestingly, we identified insertions and deletions occurring in CDR2 sequences (see Additional file 1: Figure S8). Previous analyses have shown that somatic hypermutation, rather than gene conversion, functions to increase the diversity of bovine immunoglobulin [26,50]. We extend this hypothesis by providing evidence that base insertions and deletions within bovine IgG CDR2 regions are likely operating to further diversify the bovine immunoglobulin repertoire. Similar somatic insertions/deletions outside of CDR3 have been shown to greatly alter antibody structure and function [51,52]. The results reported herein reinforce previous hypotheses regarding the presence of several mechanisms (e.g. increased CDR3 length) that serve to offset the lack of germline *V*, *D*, *J* segment diversity observed with *Bos taurus*.

### Utility of network analyses of antigen binding regions

Network-based analyses of expressed antibody repertoires provide a functional approach to visualizing antibody diversity both within and among individuals [53] and are especially useful for identifying patterns associated with antigen binding. Moreover, network topologies can be assessed statistically using measures such as *C*[41,54]. We utilized a network-based approach to visualize patterns among antigen binding region motifs of expressed bovine IgG repertoires and identified clusters within individuals (Figure 8, Additional file 1: Figures S3–S6). These clusters represent closely related antigen binding regions (CDRs1–3) as elucidated by all vs. all BLAST searches of each repertoire. Our results indicate that the expressed IgG repertoires among individuals sharing common life history traits and/or genetic backgrounds exhibit similar antigen binding networks. For example, the repertoires of crossbred USMARC Calves 1 and 2 were more common to each other than with purebred Holstein NADC Calves 3 and 4 (Figure 8). There are four distinct clusters present in the expressed IgG antigen binding regions of Calves 1 and 2 and it is possible that these individuals are 1) up-regulating antibodies as a result of exposure to a similar antigen(s), and/or 2) exhibiting preferential usage of germline *V*, *D*, *J* segment usage. The repertoires of Calves 3 and 4 do not show common clustering patterns as distinct as those shared in Calves 1 and 2; however, they are similar in that many small closely related clusters are observed in both repertoires.

Our analyses suggest that network or cluster-based approaches to characterizing expressed antibody repertoires will be useful for future studies of the immune response to pathogens, especially in controlled challenge experiments. We were able to use this approach to easily identify distinct clusters within IgG repertoires and to describe the amino acid variability observed in antigen binding regions of each cluster (Additional file 1: Figure S7). Implementation of this or similar approaches using data generated from challenge experiments will likely yield valuable information regarding the natural immune response to pathogens. We hypothesize that such information will show novel natural antigen binding solutions to specific pathogens of interest and can be used for the development of vaccines, antibody engineering, and disease surveillance initiatives.

## Conclusions

Deep sequencing of individual antibody repertoires will increase our understanding of the adaptive immune response and will be a valuable tool for a wide range of studies. We utilized CCS technology to provide baseline data of the bovine IgG repertoire. This sequencing approach results in higher per-base quality and reduces concerns about spurious results. When used in combination with network or cluster-based analyses, this approach can be used for future studies such as host immune response to infections and vaccines. Additional analyses of patterns within antigen binding sequence repertories may identify correlations between expressed antibodies and underlying genetic factors, individual life history traits, and presence or absence of pathogens.

## Abbreviations

C: Constant; *C*: Clustering coefficient; CCS: Circular consensus sequencing; CDR: Complementarity-determining region; D: Diversity germline gene segment; FR: Framework region; Ig: Immunoglobulin; J: Joining germline gene segment; NADC: National Animal Disease Center; SMRT: Single-molecule real-time; USMARC: United States Meat Animal Research Center; V: Variable; V: Variable germline gene segment; V_H_: Heavy-chain variable domain.

## Competing interests

The authors declare that they have no competing interests.

## Authors’ contributions

PAL generated and analyzed the molecular data reported herein and drafted the manuscript. TPLS conceived the study, assisted with project design and coordination, and helped to draft the manuscript. Both authors read and approved the final manuscript.

## Availability of supporting data

Supporting data are provided at the following LabArchives DOI: 10.6070/H4W66HP1.

## Supplementary Material

Additional file 1 **Figure S1.** Average number of cysteine residues within unique CDR3 sequences of differing lengths for all specimens examined. **Figure S2.** Mean position of cysteine residues within unique CDR3s of differing lengths. **Figure S3.** Complete antigen binding repertoire network for Calf 1 (6,545 nodes; 36,059 edges). **Figure S4.** Complete antigen binding repertoire network for Calf 2 (5,714 nodes; 53,715 edges). **Figure S5.** Complete antigen binding repertoire network for Calf 3 (23,996 nodes; 579,106 edges). **Figure S6.** Complete antigen binding repertoire network for Calf 4 (9,858 nodes; 118,057 edges). **Figure S7.** Amino acid content (right panels) and CDR3 lengths (left panels) of Clusters 1–4 identified in the primary antigen binding repertoire of Calf 1. **Figure S8.** Annotation of three bovine IgG sequences showing insertions/deletions within the CDR2 region (yellow). **Table S1.** Bovine IgG antigen binding regions defined using the Kabat criteria (sensu Sinclair et al. 1997 [12]) and the IMGT [9] and Paratome [32] web servers. **Table S2.** Antigen binding regions with CDR3 regions greater than 35 amino acid residues for 19 IgG molecules. Cysteine residues are shown in red to identify potential areas for disulfide bridge formations. Click here for file
