# Supplementary material for: Application of circular consensus sequencing and network analysis to characterize the bovine IgG repertoire
Source: BMC Immunol. 2012 Sep 14;13:52. doi: 10.1186/1471-2172-13-52 (PMC3500647; doi:10.1186/1471-2172-13-52)
Supplement: Additional file 1 — Figure S1. Average number of cysteine residues within unique CDR3 sequences of differing lengths for all specimens examined. Figure S2. Mean position of cysteine residues within unique CDR3s of differing lengths. Figure S3. Complete antigen binding repertoire network for Calf 1 (6,545 nodes; 36,059 edges). Figure S4. Complete antigen binding repertoire network for Calf 2 (5,714 nodes; 53,715 edges). Figure S5. Complete antigen binding repertoire network for Calf 3 (23,996 nodes; 579,106 edges). Figure S6. Complete antigen binding repertoire network for Calf 4 (9,858 nodes; 118,057 edges). Figure S7. Amino acid content (right panels) and CDR3 lengths (left panels) of Clusters 1–4 identified in the primary antigen binding repertoire of Calf 1. Figure S8. Annotation of three bovine IgG sequences showing insertions/deletions within the CDR2 region (yellow). Table S1. Bovine IgG antigen binding regions defined using the Kabat criteria (sensu Sinclair et al. 1997 [12]) and the IMGT [9] and Paratome [32] web servers. Table S2. Antigen binding regions with CDR3 regions greater than 35 amino acid residues for 19 IgG molecules. Cysteine residues are shown in red to identify potential areas for disulfide bridge formations. [file 1471-2172-13-52-S1.pdf]

Application of circular consensus sequencing and network analysis to characterize the bovine IgG repertoire

P. A. Larsen and T. P. L. Smith

Supplementary Material

Figure S1. Average number of cysteine residues within unique CDR3 sequences of differing lengths for all specimens examined.

Figure S2. Mean position of cysteine residues within unique CDR3s of differing lengths.

Figure S3. Complete antigen binding repertoire network for Calf 1 (6,545 nodes; 36,059 edges).

Figure S4. Complete antigen binding repertoire network for Calf 2 (5,714 nodes; 53,715 edges).

Figure S5. Complete antigen binding repertoire network for Calf 3 (23,996 nodes; 579,106 edges).

Figure S6. Complete antigen binding repertoire network for Calf 4 (9,858 nodes; 118,057 edges).

Figure S7. Amino acid content (right panels) and CDR3 lengths (left panels) of Clusters 1–4 identified in the primary antigen binding repertoire of Calf 1.

Figure S8. Annotation of three bovine IgG sequences showing insertions/deletions within the CDR2 region (yellow).

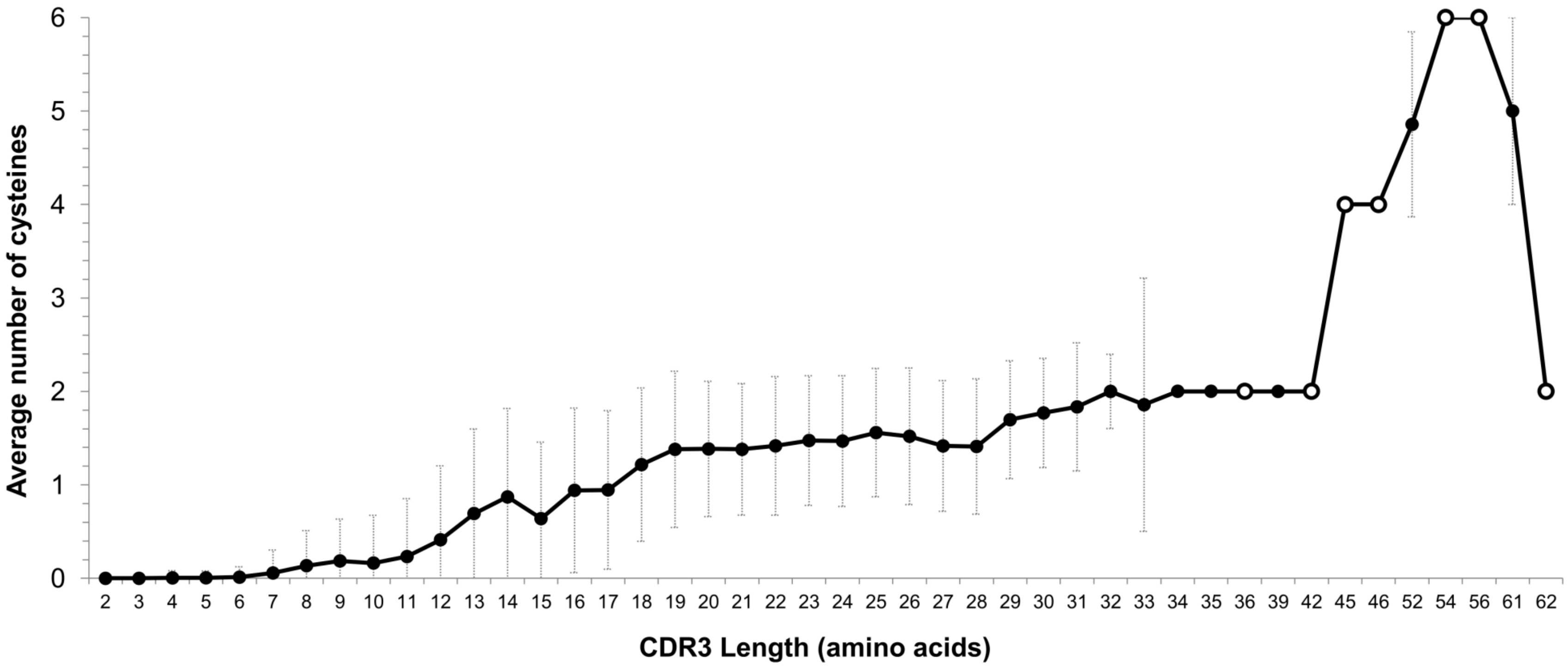
Average number of cysteines
0
1
2
3
4
5
6
2 3 4 5 6 7 8 9 10 11 12 13 14 15 16 17 18 19 20 21 22 23 24 25 26 27 28 29 30 31 32 33 34 35 36 39 42 45 46 52 54 56 61 62
CDR3 Length (amino acids)

Figure S1.

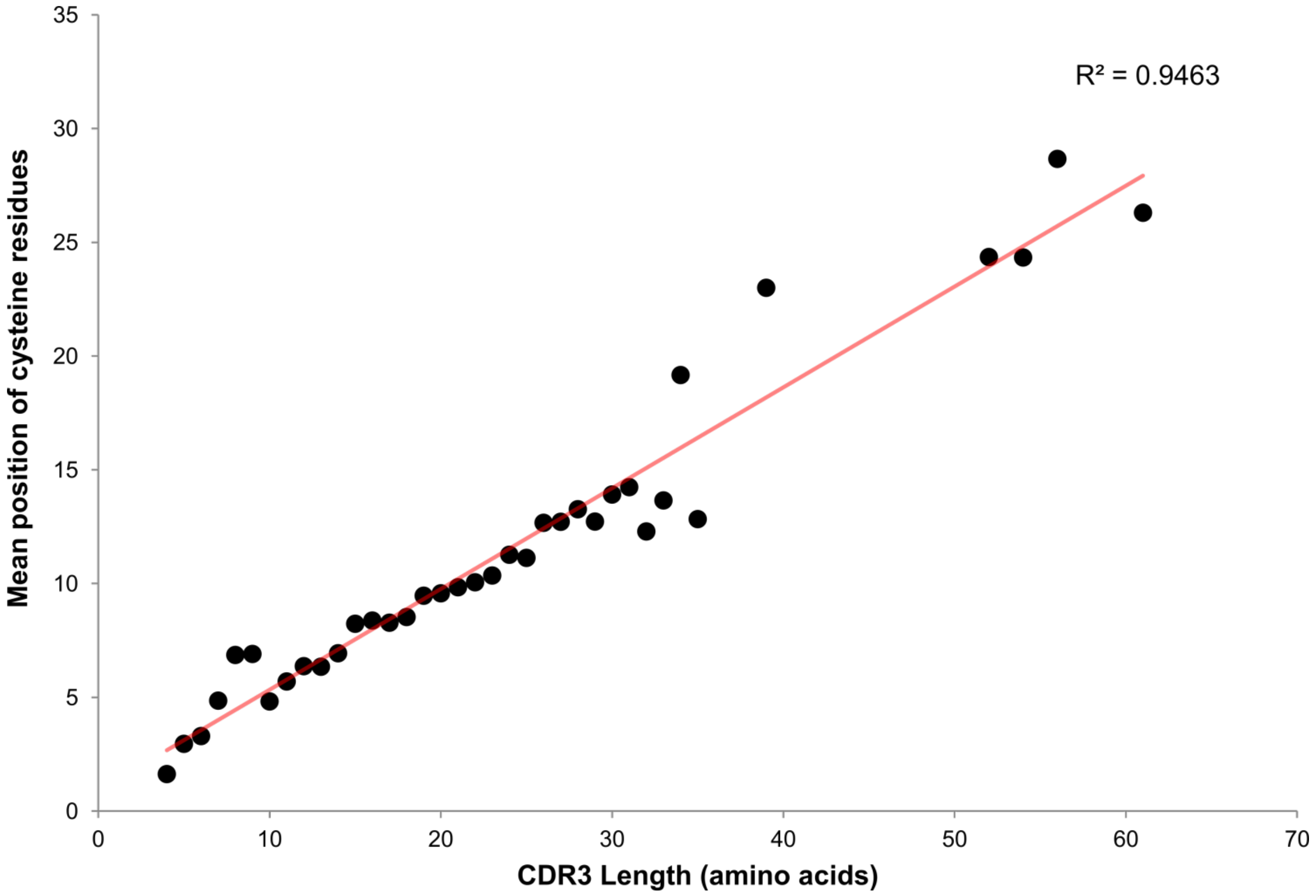

R² = 0.9463
Mean position of cysteine residues
CDR3 Length (amino acids)
0
5
10
15
20
25
30
35
0
10
20
30
40
50
60
70

Figure S2.

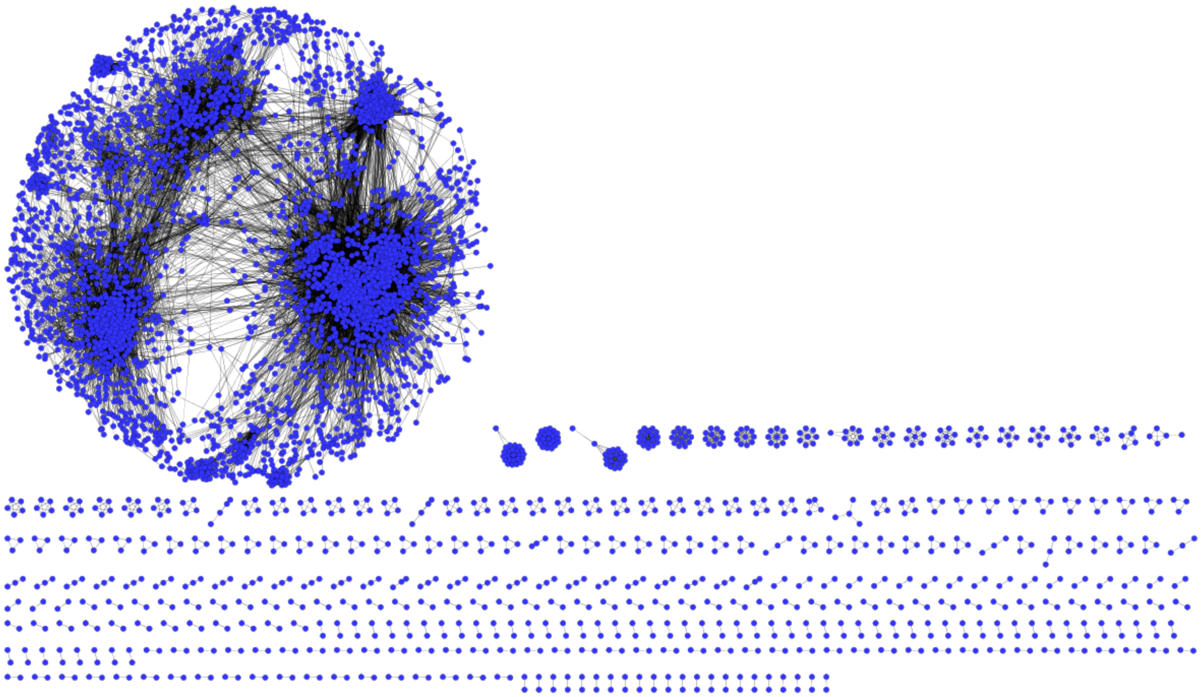

Figure S3.

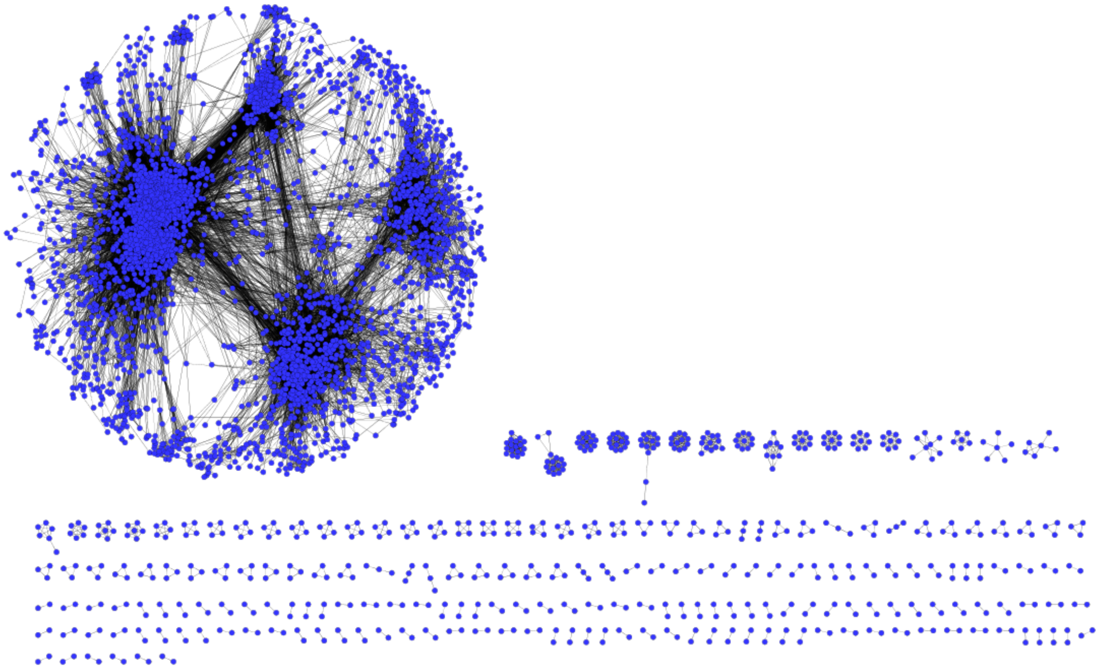

Figure S4.

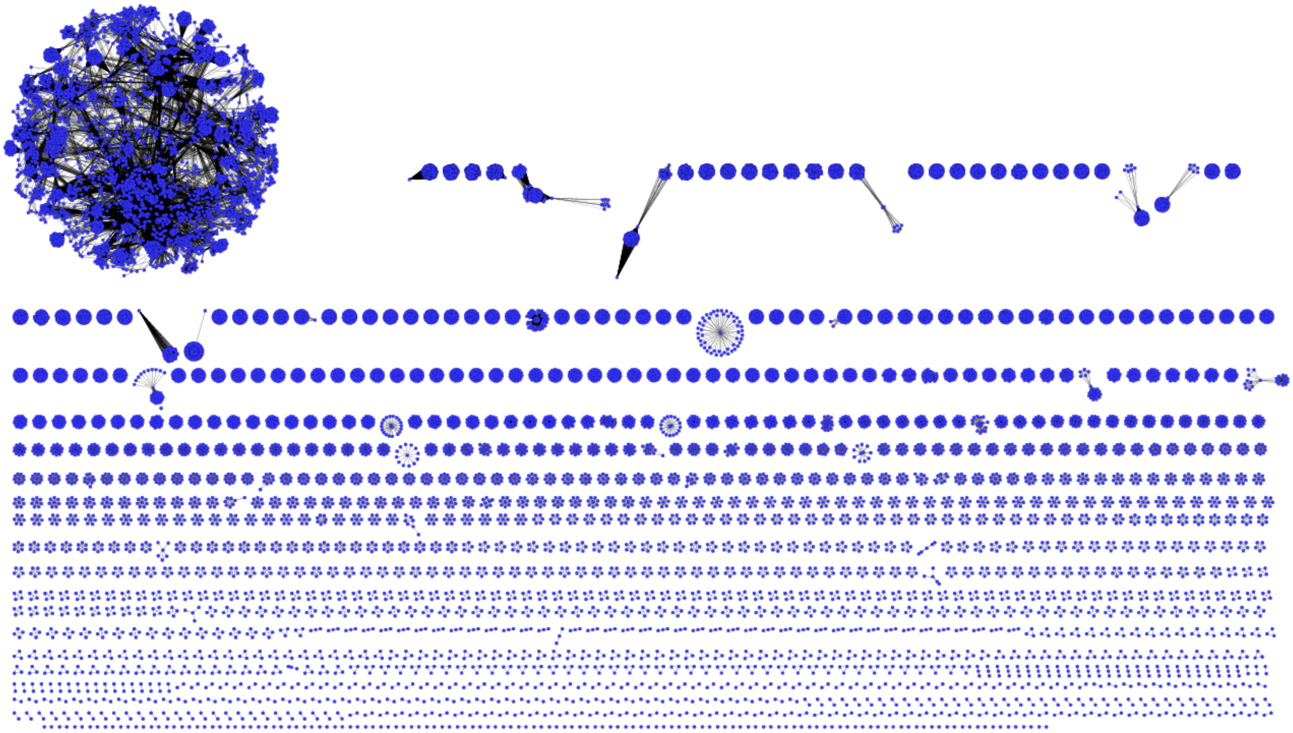

Figure S5.

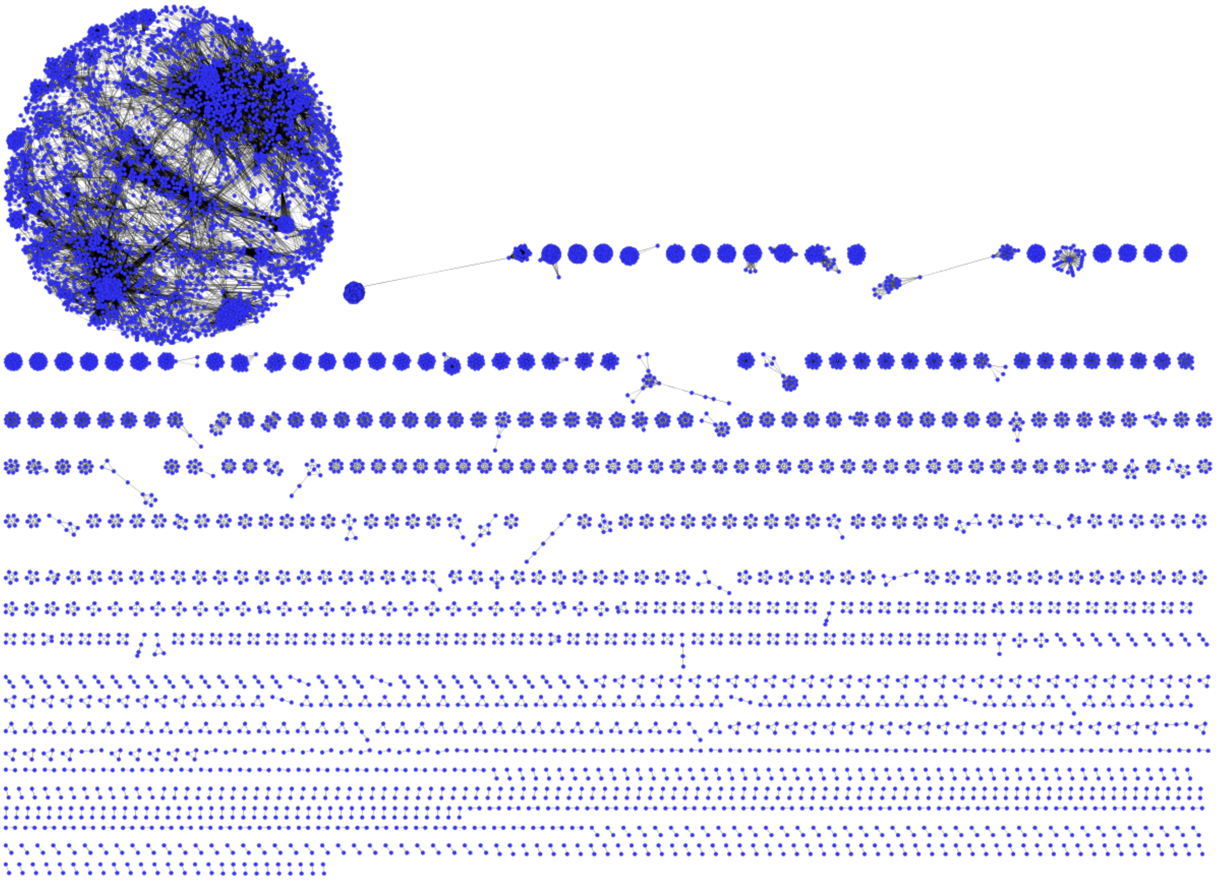

Figure S6.

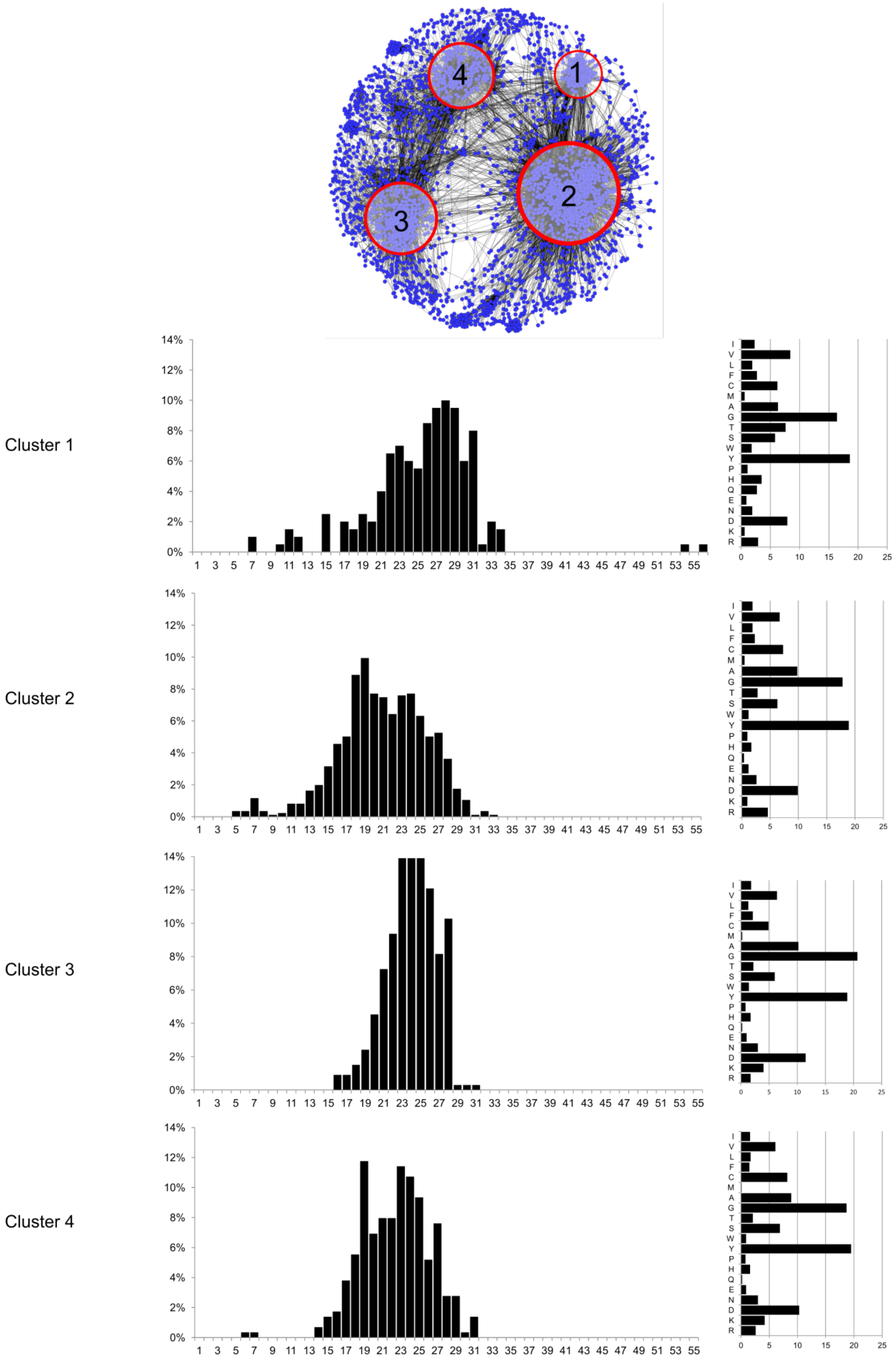
4
1
2
3
Cluster 1
Cluster 2
Cluster 3
Cluster 4
0%
2%
4%
6%
8%
10%
12%
14%
I
V
L
F
C
M
A
G
T
S
W
Y
P
H
Q
E
N
D
K
R

Figure S7.

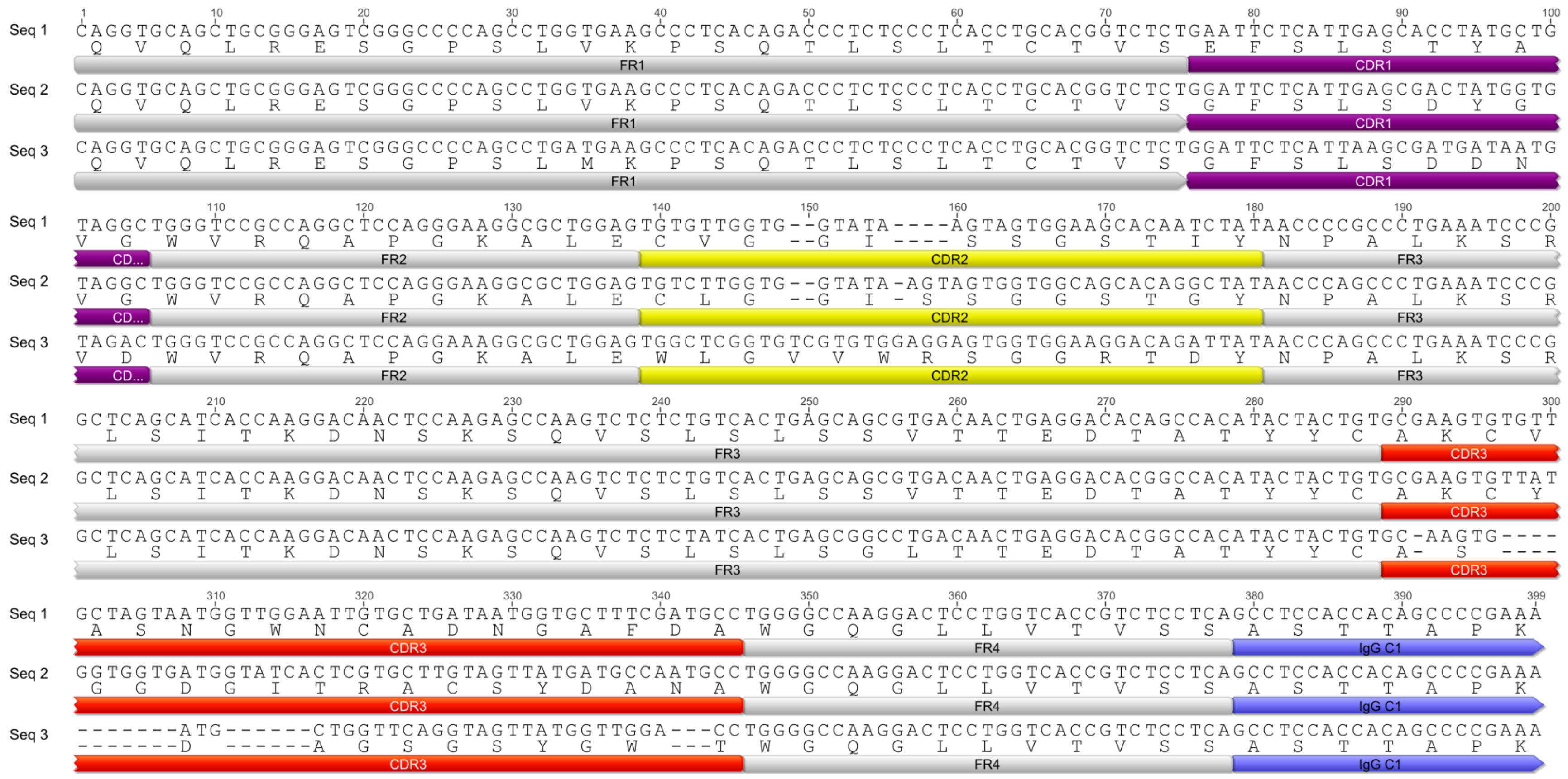

Figure S8.

Table S1. Bovine IgG antigen binding regions defined using the Kabat criteria (sensu Sinclair et al. 1997 [12]) and the IMGT [9] and Paratome [32] web servers.

Sinclair et al. 1997: U55195

CDR1 CDR2 CDR3

QVQLRESGPSLVKPSQTLSLTCTVSGFSLS**SYAVG**WVRQAPGKALEWVG**GIDGGGSTYYNPALKS**RLSITKDNSKSQVSLSVSSVTPEDTATYYCAK**CVGYAGSRSGCYYLRGGYGPHV**
DAWGQGLLVTVSSASTTAPKVYPLSS

IMGT: U55195

CDR1 CDR2 CDR3

QVQLRESGPSLVKPSQTLSLTCTVS**GFSLSSYA**VGWVRQAPGKALEWVGG**IDGGGST**YYNPALKSRLSITKDNSKSQVSLSVSSVTPEDTATYYC**AKCVGYAGSRSGCYYLRGGYGPHV**
DAWGQGLLVTVSSASTTAPKVYPLSS

Paratome: U55195

CDR1 CDR2 CDR3

QVQLRESGPSLVKPSQTLSLTCTVS**GFSLSSYAVG**WVRQAPGKALE**WVGGIDGGGSTYY**NPALKSRLSITKDNSKSQVSLSVSSVTPEDTATYYC**AKCVGYAGSRSGCYYLRGGYGPHV**
DAWGQGLLVTVSSASTTAPKVYPLSS

Sinclair et al. 1997: U55196

CDR1 CDR2 CDR3

QVQLREXGPKLVKPSQTLSLTCTTSGFSLS**SYTVG**WVRQTPGKALEWVG**GIEGDGDTCYNPALKS**RLSITKDNSKSQVSLSVSSVTPEDTATYYCAR**LTGDYVYGDGGCYAYDYNYHVD**
AWGQGLLVTVPSASTTAPKVYPLSS

IMGT: U55196

CDR1 CDR2 CDR3

QVQLREXGPKLVKPSQTLSLTCTTS**GFSLSSYT**VGWVRQTPGKALEWVGG**IEGDGDT**CYNPALKSRLSITKDNSKSQVSLSVSSVTPEDTATYYC**ARLTGDYVYGDGGCYAYDYNYHVD**
AWGQGLLVTVPSASTTAPKVYPLSS

Paratome: U55196

CDR1 CDR2 CDR3

QVQLREXGPKLVKPSQTLSLTCTTS**GFSLSSYTVG**WVRQTPGKALE**WVGGIEGDGDTCY**NPALKSRLSITKDNSKSQVSLSVSSVTPEDTATYYC**ARLTGDYVYGDGGCYAYDYNYHVD**
AWGQGLLVTVPSASTTAPKVYPLSS

Sinclair et al. 1997: U55198

CDR1 CDR2 CDR3

QVQLRESGPSLVKPSRTLSLTCTVSGFSLS**SYAVG**WVHQAPGKALECLG**GISGRGTADYNPALKS**RLSITRENSKSQVSLSLSSVTTEDTATYYCTK**STGTNADDCDDFFGDAYAYVDA**W
GQGLLVTVSSASTTAPKVYP

IMGT: U55198

CDR1 CDR2 CDR3

QVQLRESGPSLVKPSRTLSLTCTVS**GFSLSSYA**VGWVHQAPGKALECLGG**ISGRGTA**DYNPALKSRLSITRENSKSQVSLSLSSVTTEDTATYYC**TKSTGTNADDCDDFFGDAYAYVDA**W
GQGLLVTVSSASTTAPKVYP

Paratome: U55198

CDR1 CDR2 CDR3

QVQLRESGPSLVKPSRTLSLTCTVS**GFSLSSYAVG**WVHQAPGKALE**CLGGISGRGTADY**NPALKSRLSITRENSKSQVSLSLSSVTTEDTATYYCT**KSTGTNADDCDDFFGDAYAYVDA**W
GQGLLVTVSSASTTAPKVYP

Table S2. Antigen binding regions with CDR3 regions greater than 35 amino acid residues for 19 IgG molecules. Cysteine residues are shown in red to identify potential areas for disulfide bridge formations.

CDR1	CDR2	CDR3	CDR3 Length (AA)
GFSLSDKAVG	WLGGIDTGGSTGY	TTVHQPRFCGPYNYGPNGCYSDVYGYTYPNEYYVDA	36
GFSLSDKAVG	WLGGIDTGGSTGY	TVHHAKSATKTNKRDVFTCYSGYGSGCGYGYGYEFYVDA	39
GFSLSDKAVG	WLGGIDTGGSTGY	TVHHAKSATKTNKRDVFTCYSGYGSGCGYGYGYEFYVDA	39
GFSLSDKAVG	WLGGIDTGGSTGY	TVHHAKSATKTNKRDVFTCYSGYGSGCGYGYGYEFYVDA	39
GFSTSDKAVG	WLGDISSGGSTEY	TTVHQKTEKSCPGGWNDGEGCRRAFIVGRLSYTYSYEFYVHA	42
GFSLSDKAVN	WLGGIDTGGSTGY	TTVQQTCPDGYNYVYRCRVGDDCRYDCYGNHGYAYGSYFAYYVDA	45
GFSLSSYGVG	CLGGISAGGSTGYKP	TTVHQWCPDGVSYGYGCGYGYGCSGYDCYGYGGYDDGGYGGYYVDA	46
GFSLSDKAVG	WLGSVDTGGMTGY	STVHQKTTERCPDGYSYRYGCSYGDDCGYDCWDYGSRISWSYSYTYEFYVDA	52
GFSLSDKAVG	WLGSIDTGGSTGY	TTVHQKTTRNCPAGYSVHYDCSFGDGCTWTCVRHGRASSISVTYTYEWYVDA	52
GFSLSDKAVG	WLGSVDTGGMTGY	STVHQKTTERCPDGYSYRYGCSYGDDCGYDCWDYGSRISWSYSYTYEFYVDA	52
GFSLSDKAVG	WLGSIDTGGSTGY	TTVHQKTTRNCPAGYSVHYDCSFGDGCTWTCVRHGRASSISVTYTYEWYVDA	52
GFSVSSYGVN	CLGGISSSGSTAY	TTVHQTTKKSCPDGYRYGYGCGVGYCCGDYHCGLGGDSCVGEIYRHQLYVDA	52
GFSLSDKAVG	WLGSIDTGGSTGY	TTVHQTTKKSCPDGYSYGKGCGVGYCCGDYHCGLGGDSCVGEIYKHQLYVDA	52
GFSLSDKAVG	WLGSIDTGGSTGY	TTVHQTTMKRCPKGYSYGYGCGVGYCCGDWNCGLGGDSCVSEIDSYRLYVDA	52
GFSLSDKAVG	WLGSIDTDGSTGY	TTVHQTTHRSCPLGDGVGDDCGTYCCSGRSCCLYSSYDYGSYSYSYTYEWYVDA	54
GFSLSDKAVG	WLGSIDSGGNTGY	TTVHQKTNTQRSCPDGYGDGDGCGSWTCSGYDCCKYGGLGCSSYIYSYTYEFYVDA	56
GFSLSDKAVG	WLGSIDTGGSTDY	TTVHQKTKKRCPDGYSIDYRCAYGFGCNGYDCIARGGYGYGGLGADSRVRSSYSYEFDVDA	61
GFSLSDKAVG	WLGSIDTSGSTGY	TTVQQRTMKNCPDGYRHGLGCGYDYVCPGYDCCRGGGYGWGGAGGYNCASYIYAYEFYVDA	61
GFSLSDNNVG	WLGVIYSSGRANY	ARDGDGPSRPGPPDNSRRLHVHQSTPRGRRRPAAGISDACCLTVVVLIVVIFVVMVMVMVDA	62
